# CRUSADE project: Recycling technologies for ELV components to create a sustainable source of market grade materials for EU applications

**DOI:** 10.1016/j.csbj.2025.12.016

**Published:** 2025-12-24

**Authors:** Iakovos Yakoumis, Anastasia-Maria Moschovi, Olga Thoda, Asimina Katsiapi, Panagiota Stamatopoulou, Alexandros Charitos, Lesia Sandig-Predzymirska, Wambui Wamunyu, Sebeh Adjapong, Walter Murru, Francesco Veglio, Svetlana Zueva, Marco Passadoro, Antreas Afantitis, Dimitris Mintis, Andreas Tsoumanis, Eleni Papadopoulou, George Paloumpis, Nikolaos Theodoropoulos, Vasiliki Zogali, Panagiotis Xanthopoulos, Lydia Gerolymatou, Derya Dilara Ataş, Özak Durmuş, Mehmet Çelik, Naim Dönmez, Burak Güzeltepe, Yiğitcan Kama, Epameinondas Athanasakopoulos, Rosanna Babagiannou, Vincenzo Baglio, Carmelo Lo Vecchio, Irene Gatto, David Anguera Sempere, Jendrik Kromminga, Elena Santamarina, Andrés Fernández, Bjorn Gieseking, Camilo Prieto, Andrea Vincenzo Abbate, Manuel Di Frangia, Michela Spreghini, Francesco Di Muro, Diana Ferrara, Lucas Brockerhoff, Peter Weber, Philippe Lavoie, Dominique Von Sivers, Florian Witzel, Farzad Salehi, Deniz Koc, Giovanna Nicol, Renzo Costa, Flavio Parussa, Thomas Abo Atia

**Affiliations:** a Monolithos Catalysts & Recycling Limited, Athens 11476, Greece; b Institute of Nonferrous Metallurgy and Purest Materials (INEMET), TU Bergakademie Freiberg, Leipziger Str. 34, Freiberg 09599, Germany; c B.F.C. Sistemi Srl, Busnago 20040, Italy; d ENTELOS Institute Ltd, Larnaca 6059, Cyprus; e ADVENT, Patra 26504, Greece; f Sunlight Group Systimata Apothikefsis Energeias Viomichaniki Kai Emporiki Anonymi Etairia Sunlight Groupenergy Storage Systems Industrial, Athens 14564, Greece; g FORD Otomotiv Sanayi Anonim Sirketi, Istanbul 34885, Turkey; h Smart AI Solutions Single Member P.C. Smart AI Solutions Monoprosopi Idiotiki Kefalaiouchiki Etareia, Athens 13122, Greece; i Consiglio Nazionale Delle Ricerche CNR, Roma 00185, Italy; j ACCUREC-Recycling Gmbh AC, Krefeld 47809, Germany; k Asociacion De Investigacion Metalurgica Del Noroeste AIMEN, Porrino 36410, Spain; l BADGEBOX SRL, Roma 00144, Italy; m Hatch Küttner GmbH, Essen 45130, Germany; n C.R.F. S.C.p.A., Torino 10043, Italy; o Department of Chemistry, KU Leuven, Celestijnenlaan 200F, P.O. Box 2404, Leuven B3001, Belgium; p Department of Pharmacy, Frederick University, Nicosia 1036, Cyprus

**Keywords:** Hydrometallurgy, CRM, Autocatalysts, Batteries, MEAs, PCBs, Block chain, Robotic sorting

## Abstract

The European Union’s ambitions for climate neutrality and digital leadership rely on secure access to CRMs, essential for green and digital technologies but supply risks and geopolitical challenges. The CRUSADE project develops a sustainable, integrated recycling approach targeting underutilized waste streams from ELVs, including PCBs, catalytic converters, batteries and automotive fuel cells. CRUSADE proposes a universal hydrometallurgical process treating up to 500 tonnes of waste annually and recovering approximately 40 tonnes of CRMs at commercial purity. At the core of the process is a MWAL technology that enables a 15–80 % reduction in CO₂ emissions compared to conventional leaching methods, depending on feedstock composition. The process integrates AI-based automation for sorting and treatment, advanced downstream recovery and material traceability via digital tools, aligning with Industry 4.0 principles. Currently, the project is progressing toward scaling up these technologies to a modular pilot unit at TRL7, targeting 15 % faster processing time and 15 % lower product cost relative to current practices. CRUSADE aims to close material loops within the automotive and micromobility sectors, contributing to Europe’s circular economy objectives, raw material independence and climate mitigation goals under the European Green Deal.

## Highlights

• Microwave-assisted leaching improves recovery of critical raw materials (CRMs).
• AI-driven LIBS sorting enhances accuracy and process efficiency.
• Blockchain ensures traceability and regulatory compliance.
• Flexible recycling handles batteries, catalysts, MEAs, and PCBs.
• Supports EU goals for circular economy and CRM supply security.

## Graphical Abstract

**Figure:**
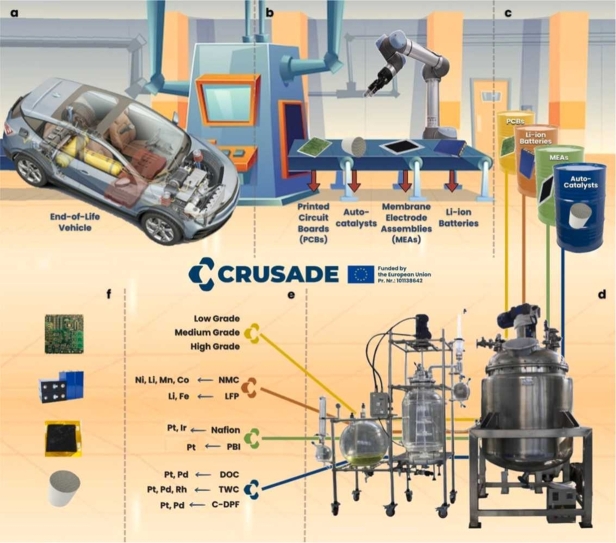

## 1 Introduction

The global transition to sustainable energy and cleaner transportation technologies has sharply increased the demand for critical raw materials (CRMs) such as platinum group metals (PGMs), lithium, cobalt, nickel and manganese. These materials are essential across multiple sectors, including automotive catalytic converters, fuel cells, batteries and electronic devices. At the same time, the increasing reliance on these resources has highlighted vulnerabilities in supply chains, environmental concerns and geopolitical risks, underscoring the need for sustainable recovery strategies. Securing a resilient and environmentally responsible supply of CRMs is therefore not only a technical challenge but also a strategic imperative for achieving climate-neutral mobility and energy solutions.

Automotive catalytic converters, for example, rely heavily on PGMs such as platinum, palladium and rhodium to reduce harmful emissions from internal combustion engines. Recycling spent autocatalysts offers a concentrated secondary source of PGMs, reducing dependency on primary mining while supporting circular economy objectives. Similarly, proton exchange membrane fuel cells contain membrane electrode assemblies (MEAs) with embedded PGMs within complex multilayer structures. Recovery of these metals is technically challenging due to the intricate assembly and the presence of polymer binders, which complicate conventional separation and extraction methods.

Electric vehicle batteries, particularly Lithium Iron Phosphate (LFP) and Nickel Manganese Cobalt (NMC) chemistries, also represent a significant reservoir of valuable CRMs. LFP batteries, while thermally stable and cost-effective, still contain lithium and other metals such as iron, aluminum and copper that require careful recovery. NMC batteries, in contrast, contain higher-value metals such as cobalt and nickel, which are economically attractive but challenging to extract due to their mixed oxide composition. Printed circuit boards (PCBs), ubiquitous in electronic devices, contain both precious metals (gold, silver, palladium) and base metals (copper, tin) embedded in multilayer fiberglass and polymer substrates, further complicating selective recovery. Conventional recycling methods, including pyrometallurgy and solvent-based hydrometallurgy, often suffer from high energy consumption, emissions of hazardous gases, low selectivity and inefficiencies in recovering dispersed metals from heterogeneous matrices.

These limitations motivate the development of innovative, integrated recycling strategies that enhance recovery efficiency, selectivity and environmental sustainability. The CRUSADE project addresses these challenges by combining advanced technologies across the recycling workflow. Key innovations include automated AI-driven sorting, high-resolution LIBS and machine vision for material identification, microwave-assisted hydrometallurgy for accelerated extraction and blockchain-enabled traceability for secure process documentation. By integrating these approaches, CRUSADE aims to maximize recovery of CRMs from end-of-life automotive components, optimize resource efficiency and contribute to a truly circular economy in the automotive and electronics sectors.

### 1.1 Objectives of the project

The CRUSADE project aims to develop and demonstrate an advanced recycling process for the recovery of CRMs from EoL vehicle components, with a focus on operational efficiency, environmental performance and industrial scalability. The core objective is to optimize and upscale a novel hydrometallurgical refining process that outperforms traditional pyro- and hydrometallurgical methods in terms of energy efficiency, emissions reduction, and cost-effectiveness. Target performance includes metal recovery rates exceeding 90 %, an 80 % reduction in CO₂ emissions, and operation at significantly lower temperatures (160–240 °C) compared to current smelting processes (>1000 °C). The process will also demonstrate the recyclability of reagents and aim for processing costs under 20 % of the market value of the recovered metals, achieving high purity levels (>95 %).

To support process optimization and traceability, the project integrates Industry 4.0 technologies, including blockchain for secure tracking of EoL components, artificial intelligence combined with LIBS (Laser-Induced Breakdown Spectroscopy), machine vision for automated sorting and material characterization and use of robotic arm for efficient sorting of the EoL components. These digital tools will enhance process adaptability, increase throughput, and support the automated control of recycling parameters based on real-time data. The targeted outcome is a fully traceable and digitally controlled pilot facility capable of processing at least 100 tonnes of EoL material annually, with a 30 % improvement in efficiency and significant gains in safety and data accuracy.

Another key focus of the project is the validation of recovered materials in real end-use applications. Recycled metals will be evaluated for their suitability in the production of catalytic converters, fuel cells, Li-ion batteries, and printed circuit boards (PCBs), with a particular emphasis on demonstrating comparable performance to virgin materials at a reduced cost (targeting ∼15 % lower). This will involve detailed characterization of purity, morphology and composition of the recycled metals, as well as development of new products using the recycled metals and benchmarking against commercial-grade products.

Furthermore, the CRUSADE concept will be validated through a pre-commercial pilot operating at Technology Readiness Level 7 (TRL 7), accompanied by the development of a comprehensive business case to ensure long-term sustainability and market deployment. This includes forecasting the availability of EoL components across the EU, establishing a pan-European collection network with a minimum annual intake of 100 tonnes, and optimizing capital and operational expenditures to remain below 70 % of the final product value. A detailed go-to-market strategy will be formulated, targeting positive cash flow within five years of pilot launch, and supported by a business plan outlining key financial metrics and commercialization pathways.

CRUSADE aims to complement existing end-of-life vehicle (ELV) dismantling and recycling efforts by targeting the recovery of valuable materials from components that are often left untreated, such as printed circuit boards (PCBs), catalytic converters, Lithium Iron Phosphate (LFP) and Nickel Manganese Cobalt (NMC) batteries, and automotive fuel cells. The goal is to extract and reuse CRMs from these components, effectively closing the loop within the automotive sector. To achieve this, the project is coordinated by MONOLITHOS Catalysts and Recycling Ltd [1] and brings together a multidisciplinary consortium covering the entire ELV value chain. This includes technology developers focused on sorting and material traceability via blockchain (AIMEN [2], BADGEBOX [3], SAISLAB [4], ENTELOS [5]); collectors and ELV providers; and leading automotive component manufacturers and recyclers (ACCUREC [6], ECORESET [7], SUNLIGHT [8], Stellantis CRF [9], Ford Otosan [10], Re-Battery [11], ADVENT [12], BFC [13], HATCH KUETTNER - KUT [14]). The consortium also includes experts in CRM recovery technologies (MONOLITHOS, KUL - KU Leuven [15], TUBAF - TU Bergakademie Freiberg [16]), as well as partners responsible for validating recovered materials in new applications (CNR [17], Ford Otosan [10]) and assessing the full pathway from ELV components to functional end products (SEC [18]). EIT RawMaterials [19] leads communication and dissemination activities, ensuring the project’s visibility and long-term impact.

## 2 Materials and methods and preliminary results - Technical innovation

### 2.1 End-of-life streams

The global transition to sustainable energy and cleaner transportation technologies has drastically increased the demand for CRMs such as PGMs, lithium, cobalt, nickel and manganese. This demand spans multiple industries, including autocatalysts, fuel cells (MEAs), Li-ion batteries and electronic wastes such as Printed Circuit Boards (PCBs). Securing a sustainable and resilient supply of these materials has become a major challenge, compounded by environmental concerns, supply chain risks and geopolitical uncertainties [20].

#### 2.1.1 Autocatalysts

Automotive catalytic converters rely heavily on PGMs such as platinum, palladium and rhodium to reduce harmful emissions from internal combustion engines. These metals are scarce, expensive and subject to volatile market fluctuations. While the automotive industry is shifting toward electrification, internal combustion engines will persist for years, sustaining the demand for autocatalysts. Recycling spent autocatalysts, an excellent secondary and concentrated source of PGMs, offers a critical pathway to recover these metals and reduce reliance on primary mining [21], [22]. Current recycling processes typically involve high-temperature smelting followed by hydrometallurgical treatment to extract metals from the ceramic or metallic substrate [23], [24], [25]. However, these methods often face limitations such as energy intensity, emission of hazardous gases, and inefficiencies in metal recovery due to the complex, heterogeneous matrix of spent catalysts [26], [27].

#### 2.1.2 Membrane Electrode Assemblies (MEAs)

MEAs, the core components of proton exchange membrane fuel cells, incorporate PGMs embedded in a complex multilayer structure composed of polymer electrolyte membranes, gas diffusion layers and catalyst layers. Recovery of PGMs and other valuable materials from EoL MEAs pose significant challenges due to the intricate cell assembly and the presence of polymeric materials that complicate separation [28], [29], [30].

Current research into MEAs recycling investigates mechanical separation, solvent dissolution and thermal treatments to selectively remove polymer binders and liberate catalyst particles. The goal is to achieve efficient PGMs recovery without degrading material properties, enabling reuse in new MEAs or other applications [31], [32]. Additionally, optimizing processes for scalability and environmental sustainability remains a critical research frontier.

#### 2.1.3 Batteries

As electric vehicles (EVs) and renewable energy storage gain momentum batteries have become a cornerstone technology enabling this transition. Among the leading chemistries in large-scale deployment are Lithium Iron Phosphate (LFP) and Nickel Manganese Cobalt (NMC) batteries. While both rely on valuable critical raw materials, they differ markedly in their material composition, performance characteristics, and the complexity of their end-of-life recovery and recycling pathways.

• **LFP batteries** use lithium iron phosphate as the cathode material, eliminating the need for cobalt and nickel. They are praised for their thermal stability, longer cycle life and lower cost but have lower energy density compared to NMC cells. LFP batteries still contain lithium and iron, along with aluminum and copper in current collectors [33].
• **NMC batteries**, on the other hand, employ a mixed cathode chemistry of nickel, manganese, and cobalt, offering higher energy density and power output, but rely on critical and expensive metals such as cobalt and nickel, which have significant supply risks and ethical concerns related to mining practices [34].

Recycling these two chemistries poses distinct challenges:

For LFP batteries, the absence of cobalt and nickel reduces the value of recovered materials but also simplifies some aspects of chemical recovery. Lithium recovery is critical, but iron recovery is less economically viable. Recycling methods must therefore focus on maximizing lithium and aluminum recovery efficiently while maintaining safety during battery dismantling and pretreatment [35], [36].

For NMC batteries, recovering high-value metals like cobalt and nickel is economically attractive but complicated by the mixed metal oxides and the need to avoid cross-contamination during chemical processes. Existing recycling methods, primarily pyrometallurgical and hydrometallurgical, are energy-intensive and risk both metal loss and hazardous waste production [37], [38].

#### 2.1.4 Printed Circuit Boards (PCBs)

PCBs are ubiquitous in electronic devices and contain significant amounts of precious metals (gold, silver, palladium), base metals (copper, tin), and rare elements. The complex multilayer structure, composed of metallic circuits embedded in fiberglass and polymer substrates, poses considerable recycling challenges [39], [40], [41]. Conventional recycling techniques include mechanical shredding, followed by pyrometallurgical and hydrometallurgical processes to recover metals. However, these often generate hazardous waste and have low recovery efficiency for precious metals due to their dispersion in complex matrices [40].

This project adopts a multidisciplinary, systems-based framework to advance the recovery of CRMs from a variety of complex waste streams, including end-of-life autocatalysts, MEAs, batteries and PCBs. At its core, the project is driven by the imperative to enhance resource efficiency and promote circularity through the development of highly selective, low-waste recovery processes tailored to the unique physical and chemical profiles of each waste type. The heterogeneity of these materials demands adaptable and integrated processing flows, making it essential to deploy flexible technologies that can accommodate varied feedstocks while minimizing environmental impact.

Key to this innovation is the integration of microwave-assisted leaching - a technology that offers significant gains in extraction kinetics and reagent efficiency. When paired with advanced sorting technologies such as laser-induced breakdown spectroscopy (LIBS), X-ray fluorescence (XRF), and AI-driven classification systems along with robotics, this approach enables rapid, accurate identification and separation of high-value fractions, improving overall process control. To ensure operational safety and efficiency, the project also emphasizes safe pretreatment methods and process intensification strategies that reduce hazards and maximize metal extraction. Additionally, embedded sustainability frameworks, including Life Cycle Assessment (LCA) and Life Cycle Costing (LCC), will guide the environmental and economic evaluation of these technologies, ensuring that proposed solutions are both scalable and aligned with circular economy principles. Through this holistic approach, the project aims to close key knowledge gaps around AI model optimization for sorting, scale-up of novel extraction technologies and the comprehensive assessment of next-generation recycling pathways.

#### 2.1.5 Collection network

The consortium established a coordinated collection network to secure EoL components for recycling demonstration, targeting materials rich in critical raw elements such as platinum group metals, lithium, cobalt and rare earths. This network enabled systematic sourcing, sorting and dispatch of diverse waste streams, including automotive catalysts, Li-ion batteries, MEAs and PCBs. Standardized handling, safe packaging and traceability protocols were implemented to ensure material integrity, proper documentation of origin and consistent assessment of component condition and composition.

So far, representative quantities of each material stream were successfully collected, encompassing both post-consumer and post-production scrap. PCBs were gathered across multiple functional categories, batteries included a range of chemistries and formats, MEAs comprised both high- and low-temperature configurations and automotive catalysts covered various types and substrate compositions. All materials were catalogued and characterized using non-destructive analytical techniques, such as XRF, to confirm the presence and composition of valuable metals.

This structured approach provided a stable and traceable feedstock for downstream recycling processes, supporting hydrometallurgical and mechanical recovery. It also enabled detailed material characterization, optimization of recovery yields and informed assessment of circular economy strategies, establishing a replicable framework for sustainable management of critical materials from complex automotive and energy-related components.

### 2.2 Sorting, preprocessing and pretreatment

#### 2.2.1 LIBS-based automated sorting - Technological concept and innovation

Within the framework of the CRUSADE initiative, AIMEN Technology Centre is developing an automated classification module that integrates laser-induced breakdown spectroscopy (LIBS) with AI-based high resolution machine vision. This dual-sensing module enables real-time identification and compositional analysis of heterogeneous components supporting selective downstream recovery processes.

The developed system combines high-resolution imaging and LIBS spectroscopy within a conveyor-based architecture, offering high flexibility to handle diverse geometries and materials typical of EoL waste streams. The AI vision subsystem performs rapid image analysis to distinguish between major component classes and subtypes based on shape, texture, and color. For the case of membrane electrode assemblies (MEAs) and automotive catalysts, subtype classification is performed through LIBS-based elemental analysis, which focuses a high-energy laser pulse onto the component to generate plasma whose emission spectrum provides a compositional fingerprint for precise material identification.

The fusion of morphological and spectroscopic data provides multi-dimensional feature sets for accurate material classification. This integrated approach represents a step-change from conventional sorting methods by enabling both high throughput and chemical specificity without the need for sample preparation. The system’s adaptability makes it suitable for a wide range of advanced material recovery workflows.

#### 2.2.2 Preliminary results and validation strategy

A compact laser source and miniaturized spectrometer were selected for implementation in the LIBS unit to ensure modularity and industrial compatibility. Laboratory-scale tests were performed to establish the measurement protocol and classification methodology. Experiments considered the variability of EoL MEAs and catalysts. Characteristic spectral emission lines of PGMs (*Pt, Pd, Rh and Ir)* were identified and optimized under various LIBS system conditions. Preliminary trials yielded classification accuracies above 65 % for reference catalysts samples.

The validated protocols and classification algorithms will next be deployed into the automated conveyor module for continuous operation testing. Future work will include quantitative benchmarking against alternative analytical methods such as XRF (time for measurement, limit of detection, sample preparation.

#### 2.2.3 Sorting of PCBs

Conventional computer-vision methods applied to PCB datasets have primarily focused on defect inspection and fault detection for manufacturing quality control rather than material valorization. Existing studies typically employ convolutional neural networks (CNNs) or hybrid image-processing pipelines to identify soldering defects, missing components, or track discontinuities in new or used boards [42], [43], [44], [45], [46], [47], [48]. Such models rely on feature extraction in the RGB domain and evaluate pixel-level anomalies to classify defective versus functional boards for industrial inspection tasks but providing no information on metallic composition. Therefore, these approaches are not directly transferable to the recycling context of the CRUSADE project where the objective is to estimate metal content of PCBs. In contrast, ENTELOS will develop an AI-based computer-vision workflow explicitly tailored for the estimation of metal content in automotive PCBs, by combining deep learning segmentation algorithms with Hue-Saturation-Value (HSV) color-space analytics and geometric descriptors to quantify metallic surface coverage associated with gold traces, connector density, and component area ratios [49], [50]. These image-derived features will subsequently be mapped to metal content classes (low, medium, high) using supervised classification models validated against experimental characterization data provided by the project partners. A curated PCB dataset will be developed, specifically designed to capture the heterogeneity of automotive PCBs obtained by the project partners and open datasets (e.g., TDD-PCB, DeepPCB, FICS-PCB) [51], [52], [53], [54]. Compared to prior literature, where deep networks such as YOLOv515 or ResNet architectures16 were trained on open datasets to localize defects at millimeter precision, the CRUSADE project will differ by optimizing the predictive mapping between optical properties and elemental composition. Within CRUSADE, a cloud-deployed inference system will be implemented to provide real-time classification of PCB images directly linked to robotic sorting and blockchain-based traceability, thereby transforming static visual inspection into a dynamic, data-driven material characterization pipeline. This presents the first documented implementation of AI-based optical estimation of metal content in PCBs for recycling applications.

#### 2.2.4 Robotic-assisted separation

Robotic-assisted separation is a promising technology to further enable the CRUSADE recycling process. While other preprocessing methods mainly consist of manual or sensor-based sorting, robotic-assisted separation integrates high-resolution sensing and manipulation with computer-controlled decision-making to accurately discriminate, handle, and route a variety of EoL materials for CRUSADE. In this approach, a robotic arm with various types of grippers and end-effectors, guided by AI-based logic systems, are utilized to pick, move, and orient diverse waste materials with sub-mm precision in CRUSADE’s system. The operations of these robotics systems are also coordinated with the data and information obtained in real-time from sensors, such as LIBS and ML vision systems, to further accurately target and sort the different waste elements and components based on their elemental compositions, surfaces, and physical properties.

Compared to conventional sorting systems, in terms of enhanced quantitative performance targets, CRUSADE's use of robotic-assisted preprocessing is expected to result in a material sorting accuracy greater than 30 % higher than that of current manual or semi-automated systems, a sorting error rate less than 5 % and a 2.5 × higher material handling throughput, as measured by parts handled per hour. In addition, by interfacing preprocessing with LIBS analytics, near real-time elemental classification of at least 95 % spectral match confidence is expected, leading to more homogeneous feedstocks delivered to hydrometallurgical processing and supporting both higher downstream recovery rates (>90 %) and lower reagent usage. A robotic sorting system can potentially better preserve the quality of high-value fractions in an EoL stream, such as PCBs, MEAs, and battery modules by avoiding mechanical damage, when working in conjunction with data-driven AI models that are trained using multimodal inputs from LIBS, RGB-D cameras and more, a robotic system is able to make more informed, selective decisions when discriminating between otherwise visually-similar, yet chemically-distinct materials such as sorting Cu-dominated regions from Ni-dominated regions on PCBs. Robotic sorting can further increase the speed of the preprocessing steps in the CRUSADE process by allowing the system to rapidly pick and place materials in a parallelized way. This info is updated in real-time from sensor data and target the most homogenous batches for feedstock uniformity and optimal leaching in downstream processing.

#### 2.2.5 Autocatalysts preprocessing

MONO has established a highly refined and systematic methodology for the preprocessing of spent automotive catalytic converters, and several limitations associated with conventional state of the art practices are addressed through its implementation. In accordance with standard workflows, the process is initiated with the collection of spent catalysts from diverse sources, including end of life vehicles and industrial waste streams.

As the first preprocessing step, a rigorous sorting procedure is carried out. Through this procedure, the spent catalytic converters are classified according to catalyst type and PGMs composition. A primary sorting stage separates the materials into three major streams: Diesel Particulate Filters (DPFs), Diesel Oxidation Catalysts (DOCs), and Three-Way Catalysts (TWCs). Each stream is subsequently refined through further subcategorization so that the feedstocks entering downstream processing are rendered as chemically uniform and compositionally well defined as possible. For Diesel Particulate Filters, a secondary sorting step differentiates between standard Diesel Particulate Filters and catalyzed Diesel Particulate Filters (cDPFs). Within the Diesel Oxidation Catalyst stream, substrates are further sorted according to their precious metal profiles, allowing components containing primarily platinum to be distinguished from those containing a platinum palladium formulation.

Following sorting, the catalytic converters are subjected to decanning, during which the metallic casings that enclose the catalytic substrates are removed. Any ancillary metallic components such as brackets or additional structural elements are also separated in order to ensure the purity of the recovered substrate material.

After decanning, crushing and milling are performed to obtain a homogeneous powdered material suitable for characterization and processing. Once milling is completed, the metal content of the samples is established by XRF analysis. Organic deposits were observed on the samples. Thus, the powdered spent catalysts were calcined in a furnace at 750 °C for 5 h so that the organic content was removed, and the thermal treatment was optimized to ensure complete decomposition of organics without altering the catalytic substrate.

Through the combination of early-stage sorting, controlled physical preprocessing, and optimized thermal treatment, an efficient and selective pathway for downstream precious metal recovery is enabled, ultimately contributing to improved process performance and resource utilization.

#### 2.2.6 MEAs preprocessing

The preprocessing steps applied to Membrane Electrode Assemblies (MEAs) are designed to separate their constituent components and enable the recovery of valuable metals from the electrocatalyst layers. MEAs contain critical metals such as platinum and iridium, which must be efficiently reclaimed. To facilitate this, catalyst powders are detached from both electrodes through targeted procedures that support effective material separation and preparation for downstream recycling.

An established process for the separation and recycling of PBI MEA components supplied by Advent was developed. These assemblies consist of gaskets, gas diffusion layers (GDLs, carbon cloth), a PBI polymeric membrane doped with phosphoric acid, and catalytic layers with a nominal loading of 1.8 mg Pt per cm² on both anode and cathode. The possible presence of additional elements such as nickel could not be excluded.

Component separation was performed through a delamination procedure that detached the MEA from the GDLs and gaskets. A solvent based method was employed to recover the electrocatalyst powder present on both the membrane and the GDLs, without dissolving the polymeric membrane. Quantification of metal loading and elemental composition was conducted using X ray fluorescence (XRF) and inductively coupled plasma optical emission spectroscopy (ICP OES). XRF analysis confirmed the presence of platinum and nickel in the recovered material.

**Fig. 1:**
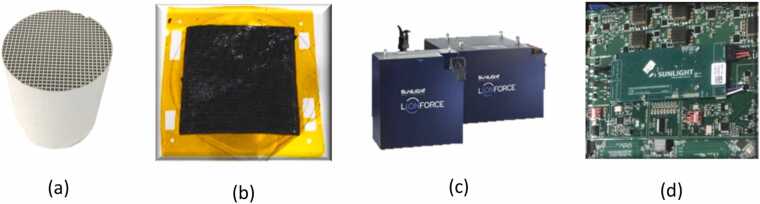
End-of-Life streams. (a) Autocatalyst, (b) MEA, (c) Batteries, (d) PCB.

**Fig. 2:**
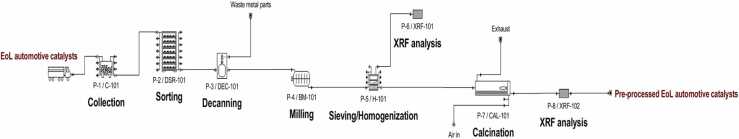
Autocatalysts preprocessing flowchart.

**Fig. 3:**
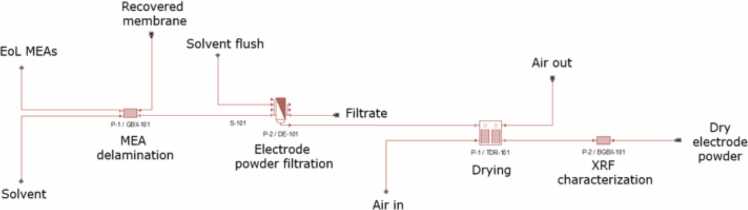
MEAs preprocessing flowchart.

**Fig. 4:**
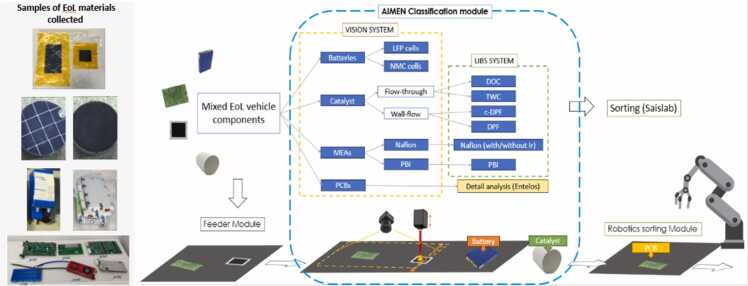
Mixed EoL components LIBS sorting; advanced material recovery workflow.

**Fig. 5:**
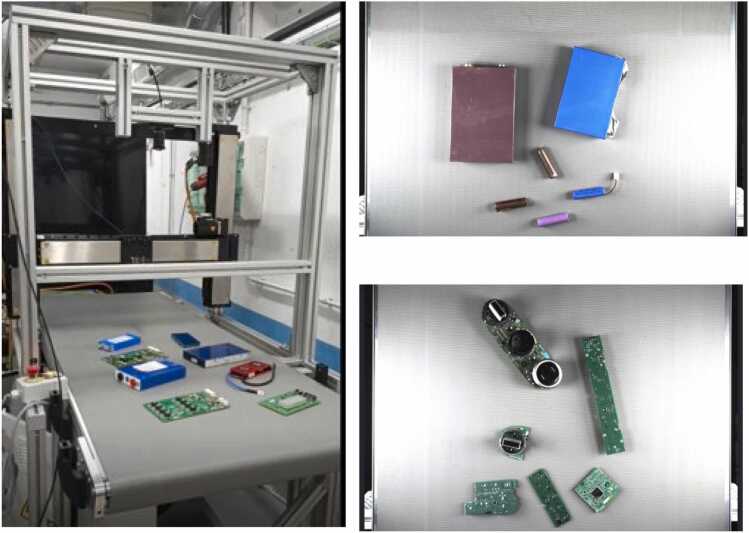
Conveyor belt prototype.

**Fig. 6:**
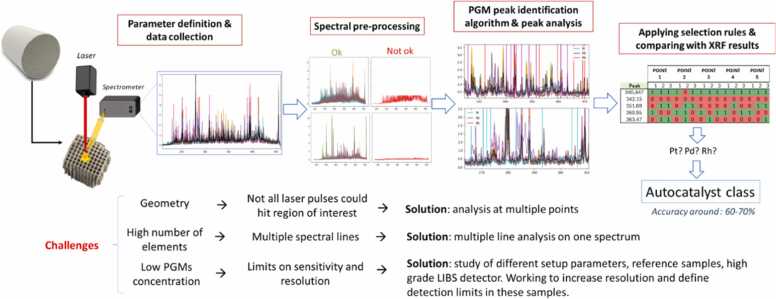
LIBS spectra highlighting emission peaks for different catalyst types.

**Fig. 7:**
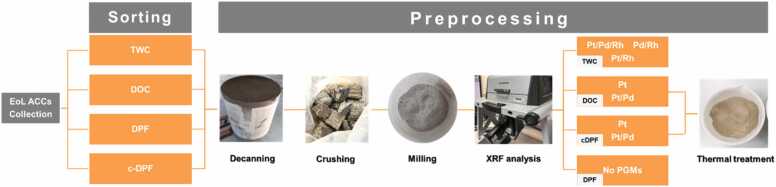
Collection and preprocessing of EoL Automotive Catalytic Converters (ACCs).

**Fig. 8:**
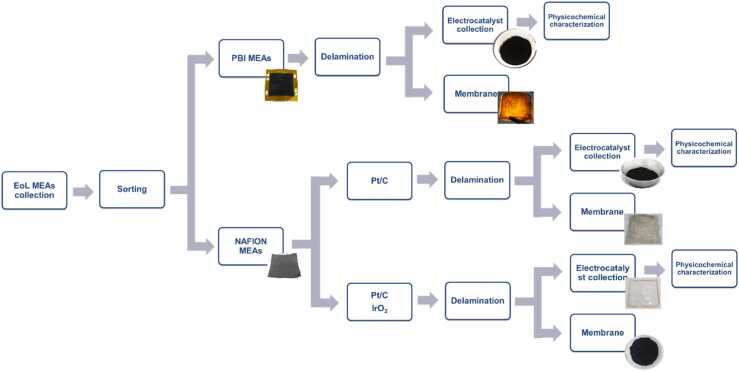
Collection and preprocessing of EoL Membrane Electrode Assemblies (MEAs).

End of life Nafion based MEAs (5–45 cm²) collected from automotive partners were processed using a similar approach. These assemblies consist of GDLs (carbon paper), a Nafion proton exchange membrane, and catalytic layers containing approximately 0.5 mg Pt per cm² at the cathode and 2 mg Ir per cm² at the anode. Separation of the Nafion membrane from the catalytic layers was achieved at room temperature using a solvent mixture. Experimental parameters such as time and temperature were adjusted to ensure that the membrane remained intact, allowing for its potential reuse. XRF analysis confirmed platinum and iridium in the recovered powders, consistent with nominal compositions. Further characterization was carried out using X ray diffraction (XRD) and scanning electron microscopy with energy dispersive X ray spectroscopy (SEM EDS) to assess structural and elemental features (Fig. 1, Fig. 2, Fig. 3, Fig. 4, Fig. 5, Fig. 6, Fig. 7, Fig. 8).

#### 2.2.7 Batteries preprocessing

**Fig. 9:**
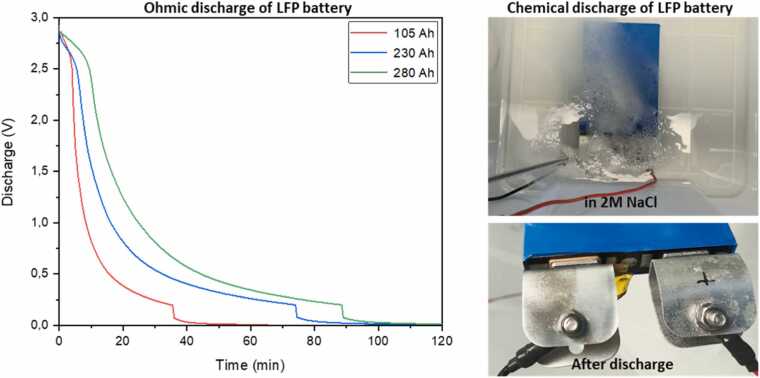
Discharging of batteries through ohmic (left) and chemical (right) methods.

To obtain LFP and NMC black masses, the batteries underwent a sequence of pre-treatment steps including discharging, dismantling, thermal treatment, and mechanical processing. Among the investigated discharging methods, ohmic discharging was found to be significantly faster and safer than chemical discharging. Three smaller cells (25, 30, and 96 Ah) were chemically discharged by immersion in NaCl solutions of varying concentrations (2 M, 3 M, and 4 M), while three larger cells (105, 230, and 280 Ah) were subjected to ohmic discharge by connecting them to a resistive load. The chemical discharge required approximately 3–6.5 h to reach a cut-off voltage of 0.6 V, whereas ohmic discharge achieved a lower cut-off voltage of 0.2 V within only 30–90 min (Fig. 9). Chemical discharging also induced corrosion of terminal connections through electrochemical reactions and posed additional safety and environmental concerns due to gas evolution and the generation of contaminated wastewater. Based on these findings, the ohmic discharge method was selected as the preferred and more sustainable approach.

In the subsequent step, two 105 Ah batteries were carefully dismantled, the electrolyte was removed, and all components were placed into sealed aluminum containers to prevent cross-contamination during thermal pre-treatment in a rotary kiln. The treatment was carried out at 600 °C for 30 min under non-oxidative conditions, aiming to decompose organics and release the active material from the metallic foils. The exhaust line was connected to the washing bottle containing 2 M NaOH and an off-gas monitoring unit to capture and quantify HF and other volatile species. As a result, pyrolysis effectively removed organic binders and solvents, enriching the residue in valuable metals such as Li, Cu, Ni, Co, Mn, and C. ACCUREC’ scalable preprocessing methodology enabled the recovery of these materials in the form of black mass, with a total mass loss of approximately 22 %, due to the decomposition of polymeric components and residual electrolytes. The pyrolyzed material exhibited improved friability, facilitating subsequent mechanical crushing and allowing the separation of black mass from coarser metallic fractions (Fe, Cu and Al flakes) by sieving.

### 2.3 Microwave-Assisted Hydrometallurgical Leaching (MWAL)

Within the CRUSADE project, the recovery process for various EoL materials was adapted to the specific characteristics of each waste stream, all incorporating a MWAL stage to enhance leaching kinetics and metal recovery yields. For all material streams (i.e., spent batteries, autocatalyst, MEAs and PCBs), a pre-treatment step was required to preconcentrate the target metals and remove non-target components. These pre-treatments included physical separation (e.g., decanting, milling, and flotation) as well as thermal or chemical conditioning to eliminate organic or other contaminants.

#### 2.3.1 PCBs Pretreatment - Pre-leaching step for base metals removal

**Fig. 10:**
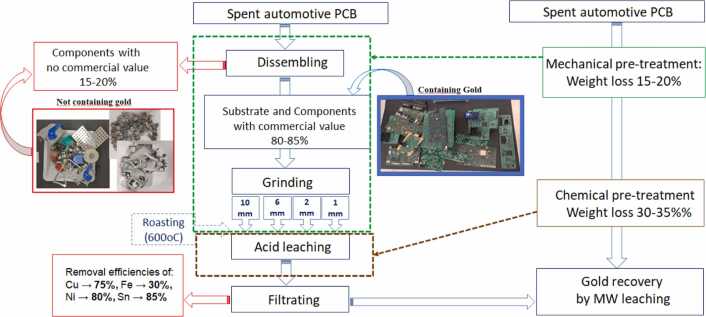
Schematic representation of the combined mechanical–chemical pretreatment process for automotive printed circuit boards (PCBs) prior to microwave leaching.

To optimize the microwave leaching process, automotive printed circuit boards (PCBs) were first subjected to a conventional acid-leaching pretreatment. The primary objective of this pretreatment was to reduce the mass of material entering the microwave leaching stage and lower the concentration of base metals that could hinder gold recovery. For this purpose, an integrated mechanical–chemical pretreatment strategy was developed and evaluated (Fig. 10).

In the first step, the PCBs were dismantled to remove components with no commercial value, reducing the total weight of the material subjected to subsequent chemical leaching by approximately 15–20 %. In the second step, the remaining PCB fractions containing precious metals underwent mechanical pretreatment through grinding, decreasing the particle size and facilitating better access to the leaching solution in gold-bearing areas. The third step of chemical pretreatment aimed at removing base metals, particularly copper. This step resulted in an additional mass loss of about 30–35 %.

The results of experiments demonstrated that the three-step pretreatment reduced the total weight of the spent PCBs by 45–55 % and enabled the removal of approximately 80 % of Cu and 90 % of Sn and Ni, improving both the cost-effectiveness and overall efficiency of the process. It highlights the potential and technological advantages of the proposed integrated mechanical–chemical pretreatment approach for enhancing gold recovery from electronic waste. The solid residue after pretreatment was farther subject for subsequent microwave leaching.

**Fig. 11:**
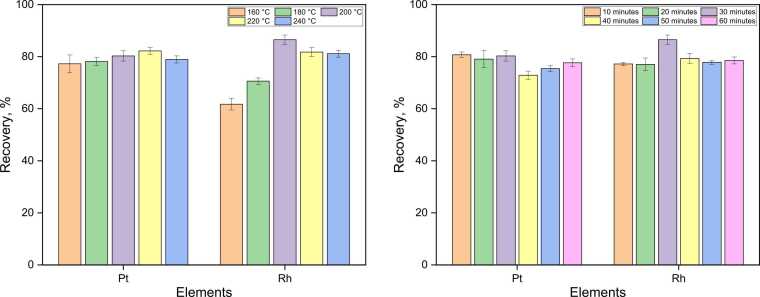
Effect of temperature (left) and time (right) on Pt and Rh leaching efficiencies during MWAL (constant parameters: 6 M HCl, 2 vol.-% H_2_O_2_, S/L ratio of 10 mg/mL, 15 min heating time, 30 min leaching time at 200 °C).

**Fig. 12:**
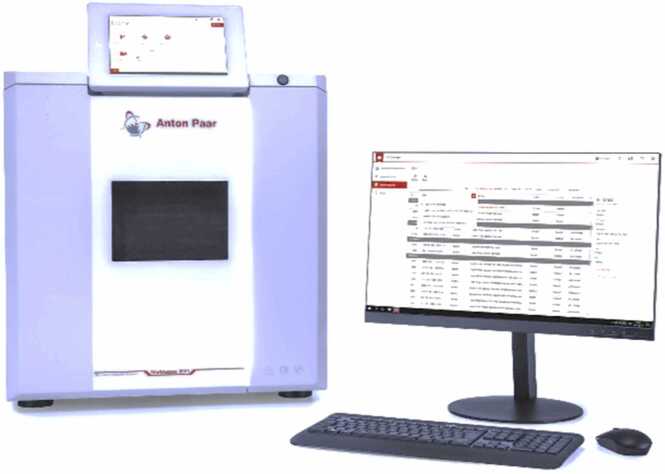
TUBAF’s Anton Paar® Multiwave 5000 system.

**Fig. 13:**
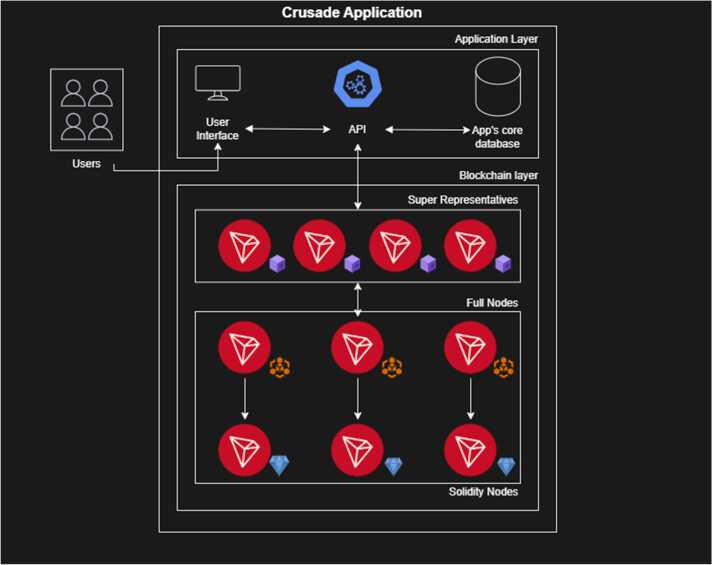
CRUSADE blockchain nodes [56].

**Fig. 14:**
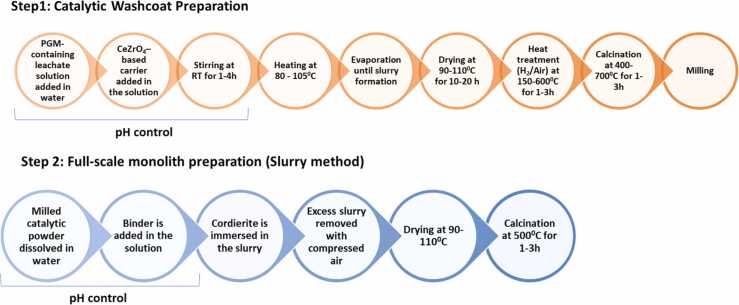
Novel synthesis route for the production of new automotive developed by MONOLITHOS.

**Fig. 15:**
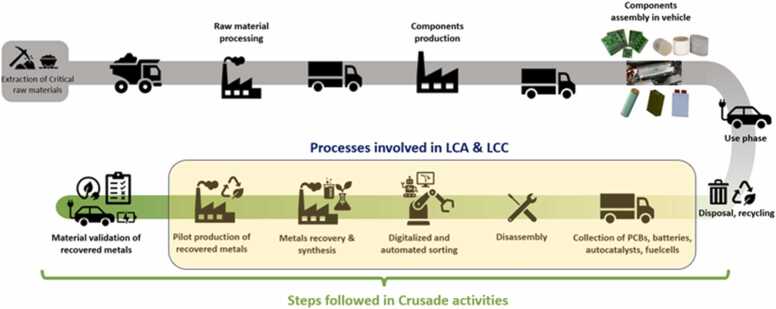
Life cycle stages and processes involved in the CRUSADE Project.

**Fig. 16:**
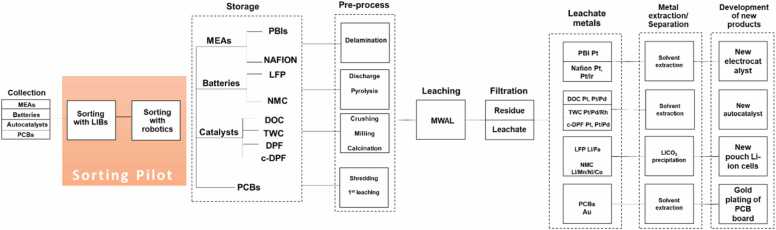
CRUSADE sequence of subtasks performed.

The core experimental work focused on optimizing the MWAL parameters - namely, reagent concentrations (HCl and H_2_O_2_), solid-to-liquid (S/L) ratio, temperature and leaching duration. For autocatalysts, first the detailed sorting protocol based on catalyst type (DPFs, c-DPFs, DOCs and TWCs) and PGM composition (Pt, Pd, and Rh), was employed followed by mechanical decanning and treatment to reduce the substrates to the fine particle size. As an example, the optimization of temperature and time for the MWAL of a three-way catalytic (TWC) converter sample, aiming at selective Pt and Rh dissolution, is shown in Fig. 11. The results indicate that the highest recovery of PGMs (80.3 ± 2.0 % of Pt and 86.5 ± 1.8 % of Rh) was achieved at 200 °C with a leaching time of 30 min. Although the process parameters have not yet been fully optimized, further adjustments could potentially yield higher recovery efficiencies. Operation at temperatures above 220 °C is not recommended, as the increased internal pressure in the microwave vessels may cause pressure release and lead to undesired PGM losses. To ensure reproducibility, five parallel experiments were performed for each condition, yielding an experimental error below 2.5 %. (Fig. 12, Fig. 13, Fig. 14, Fig. 15, Fig. 16).

Rhodium remains the most resistant element among the PGMs due to its chemical inertness and the low solubility of Rh⁰ and Rh_2_O_3_ phases under HCl–H_2_O_2_ conditions, which explains its comparatively lower leaching efficiency [57], [58]. In contrast, milder leaching conditions yielded significantly higher recovery efficiencies for the diesel oxidation catalyst (DOC) containing Pt and Pd. Using 2 M HCl with 2 H_2_O_2_ at a solid loading of 50 mg/mL (heating time 15 min followed by 30 min of leaching at 200 °C), recoveries of 91 % for Pt and 100 % for Pd were achieved.

A similar experimental strategy was applied to other types of EoL material streams, with process parameters adjusted according to the specific physicochemical characteristics of each feed and the target CRMs. Although the results are still preliminary, they clearly demonstrate the effectiveness of MWAL as a rapid and energy-efficient approach for the enhanced recovery of CRMs from complex secondary resources, with preliminary outcomes indicating PGM extraction from MEAs exceeding 85 %, and both LFP and NMC battery materials showing recovery rates above 90 %.

#### 2.3.2 Reactor design and operation (lab-scale)

Microwave-assisted leaching (MWAL) experiments were performed using an Anton Paar® Multiwave 5000 system equipped with 20 PTFE pressure vessels, each with a total capacity of 50 mL. Preliminary tests were carried out to determine the optimal leaching conditions. For each experiment, 10 mL of leaching solution (either HCl or HCl combined with H_2_O_2_) and 100–500 mg of the material was used. The samples were heated for 15 min to reach the target temperature (tested range: 120–220 °C), followed by an isothermal holding period (tested range: 5–60 min).

The Anton Paar® Multiwave 5000 enables rapid, uniform, and precisely controlled microwave heating under high-pressure conditions, ensuring reproducible leaching performance. Its ability to process up to 20 chemically resistant PTFE vessels simultaneously allows efficient parallel experimentation and significantly reduces reaction time and energy consumption. Compared to conventional thermal leaching, MWAL of EoL materials exhibits faster reaction kinetics, enhanced dissolution rates, and improved recovery of target metals (such as PGMs, Li, Co, Ni, Au and Ag), while maintaining high energy efficiency and minimizing reagent consumption.

### 2.4 Digital traceability & blockchain

#### 2.4.1 Role in process control, data integrity, compliance

Role in process control, data integrity, compliance Traceability and compliance are maintained through BADGEBOX’s TRON-based blockchain platform, which contributes to the overall digital twin system of the recycling process. This system provides an immutable real-time record of material flows, ensuring the secure tracking of EoL components from collection to final recovery.

By establishing a permanent and distributed audit trail, the platform enhances process transparency and supports regulatory compliance, which is essential for industrial partners to monitor and consult the process progress. Thanks to the structure of the TRON blockchain [55], every node in the network has a specific role, ensuring to propose a fault-tolerant system in a distributed environment.

The application is divided into two layers:

• the application’s core layer
• the blockchain layer

The application’s core layer is built on top of three services:

• the user interface (or UI) service
• the back-end service
• the app's core data layer

The UI service is the app web entry point to handle users’ interactions with the back-end service in an easy and user-friendly manner. Thanks to the UI service, users can interact with app's core configurations and monitor the processes status.

The back-end service handles users’ requests at a lower level, interacting with the app's core layer and with the blockchain layer. Additionally, the back-end service exposes a set of reusable APIs (currently used just from the UI service), which allow to plugin external services.

The blockchain layer is composed of different services, each of which has the possibility to be extended, altogether working as a whole:

• the blocks production layer
• the transactions layer
• the solidified layer

The blocks production layer is formed by Super Representative(s), each of which is responsible for producing signing transactions, packing them, producing blocks, and propagating them in the whole network.

Super Representative (or SR) node(s) range(s) from one [1] to a maximum of twenty-seven [27]. The more SRs there are, the stricter and more distributed the blockchain is. Additionally, a bigger pool of SRs ensures the system can remain operational in case of a failure of one or more nodes. The transactions layer is formed by the Full Node(s), which have, ideally, no limit of extension. This layer enables users to send transactions and query the full blockchain data, other than keeping a full copy of its ledger. In the transactions layer, every Full Node can have a Solidity Node, which is responsible to keep a copy only of the solidified blocks in its storage, allowing users to query their information in a faster and resource-friendly way.

The implementation of a blockchain-based approach is a disruptive innovation that provides a significant advantage over conventional reporting methods. It guarantees superior data integrity and enhances process adaptability and throughput. The primary objective of BADGEBOX as the technology developer is to deliver this secure technological solution for the immutable and real-time traceability of material flows. This digital integration is key to achieving a fully traceable and digitally controlled pilot facility, establishing transparent and auditable recycling value chains that align with the EU’s Green Deal objectives.

In the CRUSADE project KU Leuven is responsible for the purification and recovery of critical raw materials through advanced solvent extraction and precipitation techniques. KUL develops and optimizes solvent extraction processes to efficiently separate and purify PGMs such as palladium, platinum and rhodium from leachate solutions derived from automotive catalysts or Pt and Ir from MEAs leachates. Their work focuses on achieving high purity levels (above 98 %) using commercially available extractants and carefully designed flowsheets that ensure economic feasibility and process sustainability. Additionally, KUL refines precipitation methods to convert purified metal solutions into high-quality precursor salts suitable for gold plating of PCBs. They play a key role in delivering purified, market-ready materials that meet stringent industrial and performance requirements, enabling the circular recovery of strategic metals within the project.

### 2.5 Preparation of new products using recycled PGMs

#### 2.5.1 Autocatalysts preparation

A novel synthesis route has been developed at MONOLITHOS in which the PGM containing leachate solution is used directly as a precursor for the production of new automotive catalysts. The leachate is introduced into water without any intermediate purification, and a CeZrO_4_ based carrier is subsequently added. The mixture is stirred at room temperature for 1–4 h and then heated to 80–105 °C to ensure homogenization. Controlled evaporation is performed until slurry formation is achieved, followed by drying at 90–110 °C for 10–20 h. The dried material is then subjected to heat treatment under H₂ or air at 150–600 °C for 1–3 h, and a final calcination step is carried out at 400–700 °C for 1–3 h. The resulting solid is milled to obtain a uniform catalytic powder.

For catalyst coating, the milled powder is dispersed in water, a binder is added and cordierite monoliths are immersed in the slurry. Excess slurry is removed with compressed air, followed by drying at 90–110 °C and a final calcination at 500 °C for 1–3 h.

All synthesis and coating parameters are adjusted and optimized based on the initial composition of the leachate to ensure the correct PGM-to-carrier ratio, appropriate dispersion, and reproducible catalytic performance.

By employing the leachate solution directly, this approach eliminates conventional refinement steps, streamlines the recovery process, and reduces both material and energy demands. The method is fully aligned with circular economy principles, enabling the reintegration of recycled PGMs into high value catalytic applications. Catalysts produced through this route exhibit performance comparable to, and in some cases exceeding, that of commercial counterparts, demonstrating both the technical viability and sustainability benefits of the process.

#### 2.5.2 New MEAs development

CNR also employs a range of established synthesis methods to produce Pt/C and Pd/C catalysts, targeting noble metal loadings between 20–30 wt%, nanoparticle sizes of 3–7 nm, and electrochemically active surface areas exceeding 50 m²/g. The innovation lies not only in the use of recovered materials but in the ambition to attain benchmark-level performance typically associated with high-purity, commercially sourced PGMs. To assess their practical viability, selected catalysts derived from recovered PGMs will be integrated into 25 cm² PEM fuel cells and tested at both anode and cathode positions. Their performance will be directly compared with that of reference catalysts prepared from commercial PGM precursors under identical synthesis protocols.

#### 2.5.3 Material validation and gold recovery

CRF is actively involved in validating recovered materials by benchmarking their performance in new autocatalysts, Li-ion batteries and PCB manufacturing. This includes rigorous testing of autocatalysts and Li-ion battery cells produced from recycled materials against market standards, ensuring alignment with current regulations and automotive industry requirements. CRF also oversees the validation of gold metal recovered from the recycling of PCBs. The gold will be recovered in the form of a salt and will be used for the plating of PCB plates ensuring the recovered metal fulfils the technical requirements for industrial use. Accordingly, Ford Otosan is responsible for validating the recovered materials used in battery cathode and anode production, as well as in new DOCs.

#### 2.5.4 Life Cycle Assessment and Life Cycle Costing

LCA and LCC are being conducted by Ford Otosan to evaluate the environmental and economic performance of the recycling processes developed within the project. Both assessments follow the principles and methodological requirements of ISO 14040 and ISO 14044 to ensure transparency, comparability, and scientific rigor. The LCA includes goal and scope definition, life cycle inventory, impact assessment, and interpretation, with particular emphasis on quantifying the environmental implications of recovering CRMs from LFP and NMC batteries, PCBs, fuel cells and automotive catalysts. A gate-to-cradle system boundary is being applied to capture the environmental burdens associated with the recovery processes and the reintegration of recovered CRMs into new component manufacturing. Impact assessment is being carried out using the CML and Cumulative Energy Demand methods to analyze categories such as global warming, abiotic depletion, toxicity, and total energy demand.

In parallel, the LCC framework is being applied to assess the financial viability of the proposed hydrometallurgical and digital sorting processes. The analysis considers both capital and operational expenditures and benchmarks economic performance against conventional recycling practices. Discounting, uncertainty considerations, and sensitivity analysis are being incorporated to identify cost drivers and long-term economic hotspots. Together, the LCA and LCC approaches are enabling a combined environmental–economic evaluation of the proposed recycling routes, supporting the demonstration of reduced CO₂ emissions, improved material circularity, and competitive production costs for the recovered market-grade materials (Table 1).

**Table 1:** Comparative table of conventional recycling methods and CRUSADE approach.

Material/ Process	Conventional Methods	CRUSADE Approach	Key Advantages	Ref.
PCBs	Mechanical shredding and pyrometallurgy; often low precious metal recovery (50–70 %), high energy use, hazardous emissions	LIBS and AI sorting, pre-treatment to concentrate precious metals, targeted MW-Assisted Leaching	Higher selectivity, lower energy use, reduced hazardous waste, AI-driven automation improves throughput	[59], [60]
Automotive Catalysts	Smelting and HCl leaching; high energy, ∼80–85 % recovery	Microwave-assisted one-step hydrometallurgical leaching; lab-scale recovery > 90 %	Faster kinetics, reduced energy, high PGM recovery efficiency, scalable	[61], [62], [63]
Fuel Cells (MEAs)	Solvent extraction; ∼76 % PGM recovery; F-gases emissions	Adapted MW-assisted hydrometallurgical route, avoids toxic fluorinated byproducts; preliminary results showed PGM extraction > 85 %	Cleaner process, higher PGM selectivity, compatible with new fuel cell production	[64], [65], [66]
LFP Batteries	Pyrometallurgy or acid leaching; lithium recovery challenging, low cobalt/nickel value	MW-assisted leaching, targeted Li, Al, Cu recovery; lab-scale > 90 % extraction	Efficient lithium recovery, reduced energy consumption, safer handling	[24], [33], [35], [67]
NMC Batteries	Pyrometallurgy or mixed hydrometallurgy; risk of cross-contamination, moderate recovery efficiency	MW-assisted leaching, selective separation of Ni, Co, Mn, Li; lab-scale recovery > 90 %	High-value metal recovery, improved purity, scalable process	[37], [38], [68]
Blockchain	Paper-based and manual data reporting, delayed monitoring	Blockchain-based digital traceability; real-time monitoring	Permanent audit trail, process transparency, regulatory compliance	[69], [70]
Automation/Sorting	Manual sorting or basic sensor-based separation	Robotic arm and AI-driven sorting	High throughput, better accuracy, flexible for heterogeneous waste	[71]

## 3 Discussion

The CRUSADE project demonstrates a fully integrated, digitally enabled approach to the recovery of CRMs from complex end-of-life streams, including automotive catalysts, MEAs, Li-ion batteries, and PCBs. By combining advanced collection networks, AI-driven sorting, MWAL, solvent extraction and the development of new materials, CRUSADE addresses both the technical and environmental limitations of conventional recycling methods.

A coordinated collection network enabled the systematic sourcing of diverse EoL components, encompassing both post-consumer and post-production scrap. Standardized handling, safe packaging, and traceability protocols ensured the integrity of collected materials and provided a stable, well-characterized feedstock for downstream processes. XRF characterization confirmed the presence and concentration of CRMs, supporting process optimization and yield estimation, while establishing a framework to enhance CRM resilience and reduce reliance on primary mining.

Advanced sorting technologies, including LIBS with AI-based classification for catalysts and MEAs, as well as AI-driven computer vision for PCBs, allowed rapid, selective identification of high-value components. Preliminary lab-scale results demonstrated classification accuracies above 65 % for reference catalyst samples, with robotic-assisted separation further improving throughput, reducing mechanical damage, and delivering homogeneous feedstocks for leaching. Battery pre-treatment established that ohmic discharge is safer, faster, and more sustainable than chemical discharge, while mechanical and thermal processing steps efficiently produced black masses enriched in target metals. Autocatalysts and MEAs underwent rigorous sorting, decanning, milling and delamination, and PCBs were subjected to a three-step mechanical–chemical pretreatment that removed 45–55 % of the initial mass and 80–90 % of interfering base metals, preparing the streams for effective MWAL. Collectively, these preprocessing steps enhanced selectivity, improved recovery efficiency, and minimized environmental impact.

Microwave-assisted leaching experiments optimized reagent concentrations, temperature, solid-to-liquid ratios, and reaction times for each material stream. Preliminary results showed recovery efficiencies of 80–91 % for Pt, 86–100 % for Pd, and over 85 % for MEAs, with LFP and NMC battery materials achieving recovery rates above 90 %. Although rhodium was more resistant due to its chemical inertness, further process optimization is expected to improve its recovery. Compared to conventional leaching, MWAL offers faster kinetics, lower energy demand, and reduced reagent consumption, demonstrating both technical efficiency and environmental sustainability, and providing strong evidence for the viability of TRL7 pilot-scale implementation.

Advanced solvent extraction and precipitation processes achieved over 98 % purity for PGMs recovered from catalysts and MEAs, producing market-ready precursor salts. These high-purity materials enabled reintegration into new products, supporting both technical performance and circular economy principles. Recycled PGMs were successfully employed in the production of new automotive catalysts and PEM fuel cell catalysts, with MONOLITHOS developing a novel synthesis route using leachate solutions directly, eliminating intermediate refinement steps and reducing material and energy consumption. Catalysts produced exhibited performance comparable or superior to commercial equivalents, highlighting the technical and sustainability benefits of a closed-loop recovery approach. Recovered materials were also validated for use in Li-ion batteries and PCB manufacturing, confirming suitability for industrial applications and regulatory compliance.

Traceability and digital process control through blockchain ensured real-time, immutable tracking of material flows, enhancing transparency, regulatory compliance, and operational reliability. Preliminary life cycle assessments and life cycle costing analyses indicate significant reductions in CO₂ emissions, lower energy demand, and competitive production costs, demonstrating that the CRUSADE platform achieves measurable gains in environmental performance, CRM resilience, and circularity.

Moving forward, the project will focus on pilot-scale validation of automated sorting and purification processes, further optimization of leaching parameters for refractory metals and full-scale life cycle and economic assessments to confirm the sustainability and industrial feasibility of the approach. These efforts aim to enable the scaling of CRUSADE technologies for industrial deployment, ensuring consistent recovery of CRMs across diverse EoL streams while maintaining high environmental and economic performance.

## 4 Conclusions

The CRUSADE project delivers a disruptive, scalable, and nearly zero-waste solution for the recovery of critical raw materials (CRMs) from end-of-life (EoL) components through the integration of Industry 4.0 technologies, advanced hydrometallurgy, and AI-driven automation. To date, over 4600 kg of EoL materials, including batteries, PCBs, and automotive catalysts, are being collected and processed, providing a robust feedstock for subsequent recovery steps and pilot-scale validation.

Collected materials undergo advanced sorting and pre-treatment to maximize CRM recovery. LIBS coupled with machine learning (ML) enables selective sorting of battery and catalyst fractions, while deep learning-based PCB classification ensures high-accuracy material grading. Robotic arms and automated dismantling processes provide secure, efficient, and reproducible handling of complex EoL components, facilitating the production of feedstocks suitable for hydrometallurgical treatment. Pre-treatment strategies also include targeted leaching of PCB fractions to enhance the purity of recovered gold.

Leaching and recovery are achieved through combined chlorine-based hydrometallurgy and microwave-assisted leaching (MWAL), enabling high extraction yields under mild conditions. PGMs from automotive catalysts, MEAs, batteries, and PCBs reach total recovery levels of 90–95 %, while Li, Co, Ni, Mn, Au, Pt, and Ir are extracted at efficiencies exceeding 90 %. High-purity CRMs (>98 %) are subsequently recovered via solvent extraction and precipitation, yielding precursor salts suitable for material synthesis. Recovered metals are then valorized into new products, including Pt/C and Pd/C catalysts targeting > 1.0 W·cm⁻² peak performance in PEMFCs and LFP/NMC battery components, demonstrating performance comparable to commercial benchmarks.

Digital tools, including AI-driven process optimization, edge-cloud integration, and blockchain-based traceability, enable real-time monitoring, adaptive control, and compliance assurance throughout the workflow. Current efforts focus on finalizing pilot trials, assessing environmental and economic performance via LCA and LCC, and scaling CRUSADE technologies for industrial deployment. The project sets a strong precedent for circular, data-driven recovery of strategic materials, achieving measurable CO₂ reductions and reducing Europe’s reliance on primary CRM imports.

## Author Statement

All authors contributed to the conception and technical development of the work according to their role and stage of involvement in the project. Contributions to data generation, analysis, and manuscript preparation were distributed among the authors.

## CRediT authorship contribution statement

**Elena Santamarina:** Writing – original draft, Formal analysis. **Jendrik Kromminga:** Writing – original draft, Formal analysis. **David Anguera Sempere:** Writing – original draft, Formal analysis. **Irene Gatto:** Writing – original draft, Formal analysis. **Carmelo Lo Vecchio:** Writing – original draft, Formal analysis. **Manuel Di Frangia:** Writing – original draft, Formal analysis. **Andrea Vincenzo Abbate:** Writing – original draft, Formal analysis. **Camilo Prieto:** Writing – original draft, Formal analysis. **Bjorn Gieseking:** Writing – original draft, Formal analysis. **Andrés Fernández:** Writing – original draft, Formal analysis. **Panagiota Stamatopoulou:** Writing – review & editing, Writing – original draft, Visualization. **Asimina Katsiapi:** Writing – review & editing, Writing – original draft, Visualization, Supervision. **Olga Thoda:** Writing – review & editing, Writing – original draft, Methodology, Investigation, Data curation. **Anastasia-Maria Moschovi:** Writing – review & editing, Writing – original draft, Supervision, Investigation, Data curation, Conceptualization. **Iakovos Yakoumis:** Writing – review & editing, Writing – original draft, Supervision, Project administration, Investigation, Conceptualization. **Mehmet Çelik:** Writing – original draft, Formal analysis. **Özak Durmuş:** Writing – original draft, Formal analysis. **Derya Dilara Ataş:** Writing – original draft, Formal analysis. **Lydia Gerolymatou:** Writing – original draft, Formal analysis. **Vincenzo Baglio:** Writing – original draft, Formal analysis. **Rosanna Babagiannou:** Writing – original draft, Formal analysis. **Epameinondas Athanasakopoulos:** Writing – original draft, Formal analysis. **Yiğitcan Kama:** Writing – original draft, Formal analysis. **Burak Güzeltepe:** Writing – original draft, Formal analysis. **Naim Dönmez:** Writing – original draft, Formal analysis. **Antreas Afantitis:** Writing – original draft, Formal analysis. **Marco Passadoro:** Writing – original draft, Formal analysis. **Thomas Abo Atia:** Writing – original draft, Formal analysis. **Svetlana Zueva:** Writing – original draft, Formal analysis. **Flavio Parussa:** Writing – original draft, Formal analysis. **Renzo Costa:** Writing – original draft, Formal analysis. **Giovanna Nicol:** Writing – original draft, Formal analysis. **Panagiotis Xanthopoulos:** Writing – original draft, Formal analysis. **Vasiliki Zogali:** Writing – original draft, Data curation. **Nikolaos Theodoropoulos:** Writing – original draft, Formal analysis. **George Paloumpis:** Writing – original draft, Formal analysis. **Eleni Papadopoulou:** Writing – original draft, Formal analysis. **Andreas Tsoumanis:** Writing – original draft, Data curation. **Dimitris Mintis:** Writing – original draft, Data curation. **Philippe Lavoie:** Writing – original draft, Formal analysis. **Peter Weber:** Writing – original draft, Formal analysis. **Lucas Brockerhoff:** Writing – original draft, Formal analysis. **Francesco Veglio:** Writing – original draft, Formal analysis. **Diana Ferrara:** Writing – original draft, Formal analysis. **Walter Murru:** Writing – original draft, Formal analysis. **Francesco Di Muro:** Writing – original draft, Formal analysis. **Michela Spreghini:** Writing – original draft, Formal analysis. **Wambui Wamunyu:** Writing – original draft, Formal analysis. **Lesia Sandig-Predzymirska:** Writing – original draft, Formal analysis. **Alexandros Charitos:** Writing – original draft, Formal analysis. **Deniz Koc:** Writing – original draft, Formal analysis. **Farzad Salehi:** Writing – original draft, Formal analysis. **Florian Witzel:** Writing – original draft, Formal analysis. **Dominique Von Sivers:** Writing – original draft, Formal analysis. **Sebeh Adjapong:** Writing – original draft, Formal analysis.

## Declaration of Competing Interest

The authors declare that there are no conflicts of interest associated with this manuscript.

## References

[1] MONOLITHOS Catalysts Ltd. 〈www.innovation.monolithos.gr〉.

[2] AIMEN. 〈www.aimen.es/en〉.

[3] Badgebox. 〈www.badgebox.com〉.

[4] SAISLAB. 〈www.saislab.com〉.

[5] ENTELOS Institute. 〈www.entelos.eu〉.

[6] ACCUREC. 〈www.accurec.de〉.

[7] ECORESET. 〈www.ecoreset.gr〉.

[8] SUNLIGHT. 〈www.sunlight-group.com〉.

[bib9] 9CRF. it.wikipedia.org/wiki/Centro_Ricerche_Fiat.

[10] Ford Otosan. 〈www.fordotosan.com.tr〉.

[11] Re-Battery. 〈www.rebattery.gr〉.

[12] ADVENT. 〈www.advent.energy〉.

[13] BFC Sistemi. 〈www.bfcsistemi.it〉.

[14] HATCH KUTTNER. 〈www.kuettner.com〉.

[15] KU Leuven. 〈www.kuleuven.be〉.

[16] TU Bergakademie Freiberg. 〈www.tu-freiberg.de〉.

[17] CNR. 〈www.cnr.it〉.

[18] SE&C. 〈www.senc.gr〉.

[19] EIT RawMaterials. 〈www.eitrawmaterials.eu〉.

[20] Crîstiu D., d’Amore F., Bezzo F. (2025). Optimal design of sustainable supply chains for critical raw materials recycling in renewable energy technologies. Resour Conserv Recycl.

[21] Yang D., Yang Q., Ma W., Ma X., Wang S., Lei Y. (2023). Characteristics of spent automotive catalytic converters and their effects on recycling platinum-group-metals and rare-earth-elements. Sep Purif Technol.

[22] Yakoumis I., Panou M., Moschovi A.M., Panias D. (2021). Recovery of platinum group metals from spent automotive catalysts: A review. Clean Eng Technol.

[23] Papagianni S., Moschovi A.-M., Polyzou E., Yakoumis I. (2022). Platinum recovered from automotive heavy-duty diesel engine exhaust systems in hydrometallurgical operation. Metals.

[24] Deng H., Wang B., Xu J., Yang G., Shi Z., Zhu H. (2025). A comprehensive review of whole process typical hydrometallurgical technologies for recycling waste lithium-ion batteries. Sep Purif Technol.

[25] Saguru C., Ndlovu S., Moropeng D. (2018). A review of recent studies into hydrometallurgical methods for recovering PGMs from used catalytic converters. Hydrometallurgy.

[26] Ali H., Khan H.A., Pecht M. (2022). Preprocessing of spent lithium-ion batteries for recycling: Need, methods, and trends. Renew Sustain Energy Rev.

[27] Elgarahy A.M., Eloffy M.G., Priya A.K., Hammad A., Zahran M., Maged A. (2024). Revitalizing the circular economy: an exploration of e-waste recycling approaches in a technological epoch. Sustain Chem Environ.

[28] Alipour Moghaddam J., Parnian M.J., Rowshanzamir S. (2018). Preparation, characterization, and electrochemical properties investigation of recycled proton exchange membrane for fuel cell applications. Energy.

[29] Robert M., Dubelley F., Paul A., Svecova L., Bas C. (2025). Investigation of membrane–electrode separation processes for the recycling of ionomer membranes in end-of-life PEM fuel cells. Energy Fuels.

[30] Arun M., Giddey S., Joseph P., Dhawale D.S. (2025). Challenges and mitigation strategies for general failure and degradation in polymer electrolyte membrane-based fuel cells and electrolysers. J Mater Chem A.

[31] Duclos L., Lupsea M., Mandil G., Svecova L., Thivel P.-X., Laforest V. (2017). Environmental assessment of proton exchange membrane fuel cell platinum catalyst recycling. J Clean Prod.

[32] Khalili M., Harameen H.M.A., Choi B., Bae M., Lee H., Kim S.-K. (2025). Sustainable PGM recovery processes for fuel cell and electrolyzer applications. ACS Appl Energy Mater.

[33] Zhao T., Mahandra H., Marthi R., Ji X., Zhao W., Chae S. (2024). An overview on the life cycle of lithium iron phosphate: synthesis, modification, application, and recycling. Chem Eng J.

[34] Li, Y.; Omar, N.; Nanini-Maury, E.; Bossche, P.Van den; Mierlo, J.Van. Performance and Reliability Assessment of NMC Lithium Ion Batteries for Stationary Application. In *2016 IEEE Vehicle Power and Propulsion Conference (VPPC)*; 2016; pp 1–6. doi:10.1109/VPPC.2016.7791747.

[35] Zhang M., Wang L., Wang S., Ma T., Jia F., Zhan C. (2023). A Critical Review on the Recycling Strategy of Lithium Iron Phosphate from Electric Vehicles. Small Methods.

[36] Xu P., Tan D.H.S., Jiao B., Gao H., Yu X., Chen Z. (2023). A Materials Perspective on Direct Recycling of Lithium-Ion Batteries: Principles, Challenges and Opportunities. Adv Funct Mater.

[37] Davis K., Demopoulos G.P. (2023). Hydrometallurgical recycling technologies for NMC Li-ion battery cathodes: current industrial practice and new R&D trends. RSC Sustain.

[38] Winkowska-Struzik M., Buchberger D., Uhrynowski W., Struzik M., Boczar M., Rogulski Z. (2024). From NMC to NMC – Challenges of Direct Recycling. ECS Meet Abstr.

[39] Awasthi Abhishek Kumar, Zlamparet Gabriel Ionut, Zeng Xianlai, Li Jinhui (2017). Evaluating waste printed circuit boards recycling: Opportunities and challenges, a mini review. Waste Manag Res.

[40] Mir S., Dhawan N. (2022). A comprehensive review on the recycling of discarded printed circuit boards for resource recovery. Resour Conserv Recycl.

[41] Zhao W., Xu J., Fei W., Liu Z., He W., Li G. (2023). The reuse of electronic components from waste printed circuit boards: a critical review. Environmental Science Advances.

[42] Sharma H., Kumar H. (2024). A computer vision-based system for real-time component identification from waste printed circuit boards. J Environ Manag.

[43] Hu B., Wang J. (2020). Detection of PCB surface defects with improved faster-RCNN and feature pyramid network. IEEE Access.

[44] Adibhatla V.A., Chih H.-C., Hsu C.-C., Cheng J., Abbod M.F., Shieh J.-S. (2020). Defect detection in printed circuit boards using you-only-look-once convolutional neural networks. Electronics.

[45] Alghassab M.A. (2022). Defect detection in printed circuit boards with pre-trained feature extraction methodology with convolution neural networks. Comput Mater Contin.

[46] Chen X., Wu Y., He X., Ming W. (2023). A comprehensive review of deep learning-based PCB defect detection. IEEE Access.

[47] Chaudhary, V.; Dave, I.R.; Upla, K.P. Automatic visual inspection of printed circuit board for defect detection and classification. In 2017 International Conference on Wireless Communications, Signal Processing and Networking (WiSPNET); 2017; pp 732–737. doi:10.1109/WiSPNET.2017.8299858.

[48] Ling Q., Isa N.A.M. (2023). Printed Circuit Board Defect Detection Methods Based on Image Processing, Machine Learning and Deep Learning: A Survey. IEEE Access.

[49] Kirillov, A.; Mintun, E.; Ravi, N.; Mao, H.; Rolland, C.; Gustafson, L.; et al. Segment Anything. In 2023 IEEE/CVF International Conference on Computer Vision (ICCV); 2023; pp 3992–4003. doi:10.1109/ICCV51070.2023.00371.

[50] Roy Anirban, Todorovic S., Leibe Bastian, Matas J., S. N., W. M. (2016). Computer Vision – ECCV 2016.

[51] Lu H., Mehta D., Paradis O.P., Asadizanjani N., Tehranipoor M.M., Woodard D. (2020). FICS-PCB: A Multi-Modal Image Dataset for Automated Printed Circuit Board Visual Inspection. IACR Cryptol EPrint Arch.

[52] Tang, S.; He, F.; Huang, X.; Yang, J. Online PCB Defect Detector On A New PCB Defect Dataset. ArXiv 2019, abs/1902.06197.

[53] Ding R., Dai L., Li G., Liu H. (2019). TDD-net: a tiny defect detection network for printed circuit boards. CAAI Trans Intell Technol.

[54] Pramerdorfer C., Kampel M. (2015). A dataset for computer-vision-based PCB analysis. 2015 14th IAPR Int Conf Mach Vis Appl (MVA).

[55] TRON - DECENTRALIZE THE WEB. 〈https://tron.network/〉. 〈https://tron.network/〉 Accessed 25.12.09.

[56] CRUSADE Application. 〈https://market.badgebox.com/crusade/app/login〉. 〈https://market.badgebox.com/crusade/app/login〉 Accessed 25.12.09.

[57] Yakoumis I., Moschovi A., Panou M., Panias D. (2020). Single-Step Hydrometallurgical Method for the Platinum Group Metals Leaching from Commercial Spent Automotive Catalysts. J Sustain Metall.

[58] Abo Atia T., Wouters W., Monforte G., Spooren J. (2021). Microwave chloride leaching of valuable elements from spent automotive catalysts: Understanding the role of hydrogen peroxide. Resour Conserv Recycl.

[59] Akcil A., Erust C., Gahan C.S., Ozgun M., Sahin M., Tuncuk A. (2015). Precious metal recovery from waste printed circuit boards using cyanide and non-cyanide lixiviants – A review. Waste Manag.

[60] Cui J., Zhang L. (2008). Metallurgical recovery of metals from electronic waste: a review. J Hazard Mater.

[61] Lanaridi O., Sahoo A.R., Limbeck A., Naghdi S., Eder D., Eitenberger E. (2021). Toward the Recovery of Platinum Group Metals from a Spent Automotive Catalyst with Supported Ionic Liquid Phases. ACS Sustain Chem Eng.

[62] Karim S., Ting Y.-P. (2021). Recycling pathways for platinum group metals from spent automotive catalyst: a review on conventional approaches and bio-processes. Resour Conserv Recycl.

[63] Trinh H.B., Lee J., Suh Y., Lee J. (2020). A review on the recycling processes of spent auto-catalysts: Towards the development of sustainable metallurgy. Waste Manag.

[64] Wittstock R., Pehlken A., Wark M. (2016). Challenges in automotive fuel cells recycling. Recycling.

[65] Simons A., Bauer C. (2015). A life-cycle perspective on automotive fuel cells. Appl Energy.

[66] Xu F., Mu S., Pan M. (2010). Recycling of membrane electrode assembly of PEMFC by acid processing. Int J Hydrog Energy.

[67] Ding Y., Fu J., Zhang S., He X., Zhao B., Ren J. (2024). Advances in recycling LiFePO4 from spent lithium batteries: A critical review. Sep Purif Technol.

[68] Fan J., Luo H., Wang T., Dai S. (2024). Progress in direct recycling of spent lithium nickel manganese cobalt oxide (NMC) cathodes. Energy Storage Mater.

[69] Bułkowska K., Zielińska M., Bułkowski M. (June 1, 2024). Energies.

[70] Jiang P., Zhang L., You S., Fan Y.Van, Tan R.R., Klemeš J.J. (2023). Blockchain technology applications in waste management: Overview, challenges and opportunities. J Clean Prod.

[71] Aschenbrenner D., Colloseus C., Khoury R., Fangerow N. (2023). Robot-assisted automated sorting techniques for plastic recycling. Procedia CIRP.

